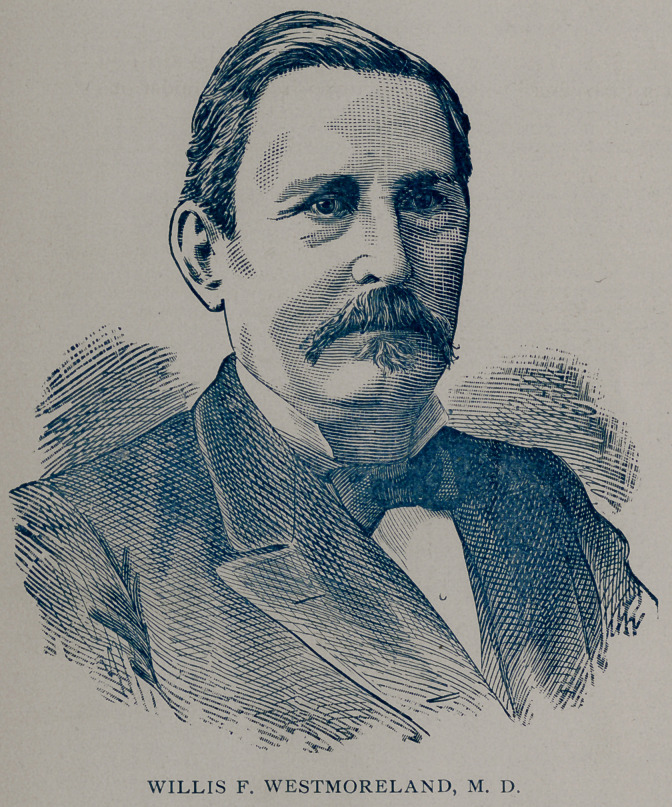# Obituary

**Published:** 1890-06

**Authors:** 


WILLIS F. WESTMORELAND, M. D.

### OBITUARY.

### DR. WILLIS F. WESTMORELAND.

With sorrowing hearts we humbly bow before the sad and mysterious Providence which has removed from our midst one of Georgia's noblest and most useful sons. Dr. Willis F. Westmoreland is no more. Truly a great man has fallen; he has gone to his reward; his course is finished; his grand career is ended, but his name will go sounding down the ages as friend, benefactor, physician and surgeon. He was born in Georgia, June 1st, 1828, and departed this life June 27th, 1890. When quite young he chose the medical profession for his vocation, and attended his first course in Augusta, Ga., in what is now known as the Medical Department of the University of Georgia.

He took the degree of M. D. in the Jefferson Medicel College at Philadelphia. He then graduated in the Medical Department of the University of Nashville, and went to Paris, where he remained for a considerable time, devoting himself to the study of surgery. He came back to Atlanta, and, with his brother, Dr. John G. Westmoreland, founded the Atlanta Medical College, in which he was appointed to the chair of Surgery, which position he occupied with distinguished ability for thirty-six years.

It is also proper to state that Dr. Westmoreland made a second trip to Europe, and returned in 1855, when the *Atlanta Medical and Surgical Journal* was established, of which he became one of the editors. He was surgeon in the Confederate army, and was for three years in charge of the Medical College Hospital in Atlanta. He was a man of indefatigable energy, and rose to well-merited distinction because of his candor, his humanity and his well known skill and ability. There was nothing in general surgery that he could not do. He wielded the knife with a dexterity unsurpassed by any American surgeon. His judgment was sure, and his courage never wavered in the least. As a diagnostician he had but few equals. In the great domain of surgery he sought no particular field. He was abreast with the times. As a counsellor he was safe, as a practitioner and operator cautious and conservative. He was a skillful physician, but surgery claimed and appreciated him. As a teacher, he was plain, clear and impressive. No professor was more dreaded in the green room, and still he was very popular with the classes. He greatly loved the profession, and educated his only son for it, that he might have a worthy successor of his own name. This was the dream of his life. As physician to the State penitentiary, he wrought reforms, systematized the medical department, established the best sanitary regulations, and produced the finest health reports ever known.

Dr. Westmoreland was unswerving in his fidelity to every trust; was a public spirited citizen; true to his friends and magnanimous to his enemies. He exercised a broad charity, which only belongs to great minds.

But he is gone, to mingle no more with loving friends on earth. The deft hand of the great surgoon rests from labor. The eye so quick to see disease is closed in death. The heart so generous, so pure, so full of love for mankind, has ceased its throbbings forever. "*Requiescat in pace.*"

## Figures and Tables

**Figure f1:**